# Future Prospects for the Development of Cost-Effective Adenovirus Vaccines

**DOI:** 10.3390/ijms18040686

**Published:** 2017-03-23

**Authors:** Cyrielle Fougeroux, Peter J. Holst

**Affiliations:** Department of Immunology and Microbiology, Copenhagen University, København K 1014, Denmark; cyrielle@sund.ku.dk

**Keywords:** adenovirus, vaccine, replication competent, transgene, affordable vaccine candidates, protective immunity, vaccine technology platform, immune responses

## Abstract

Vaccination is one of the most efficient tools for disease prevention, and a continuously growing field of research. However, despite progress, we still need more efficient and cost-effective vaccines that would improve access to those in need. In this review, we will describe the status of virus-vectored vaccine technology with a focus on adenoviral-based vaccines. Adenovirus (Ad) vaccines have proven to be efficient in military vaccinations against Ad4 and Ad7 and as highly efficient vectored vaccines against rabies. The question of how other adenovirus-based vaccines can become as efficient as the rabies vaccine is the underlying theme in this review. Here, we will first give an overview of the basic properties of vectored vaccines, followed by an introduction to the characteristics of adenoviral vectors and previously tested modifications of the vector backbone and expression cassettes, with a focus on how they can contribute to increased vaccine cost-effectiveness. Finally, we will highlight a few successful examples of research that have attempted to improve the use of adenoviral-based vaccines by improving the transgene immunogenicity.

## 1. Introduction

Vaccination has greatly reduced the burden of infectious diseases over the last 200 years, and clean water is the only health intervention proven to perform better than vaccination to overcome sickness and death [1]. Furthermore, vaccines have become of increasingly better quality and effectiveness [2], and they hold a considerable safety advantage as compared to therapeutic medicines [3].

Despite these promising advancements, there is still a need for more cost-effective and also long-lasting vaccine platforms. This is required in order to facilitate distribution to developing countries and to facilitate veterinary prevention of infectious diseases, as opposed to antibiotic treatment or mass culling when faced with serious infectious diseases.

The very first vaccine was the cowpox virus that was naturally attenuated in humans and could provide cross-protective immunity against smallpox. This vaccine was dependent on the fortunate availability of a related virus, but was highly effective and was the principal necessary tool in the eradication of smallpox. After the discovery of vaccination with cowpox, Pasteur provided the more generalizable principle of vaccination when he discovered that attenuation or inactivation of a pathogen (in his case, anthrax and later rabies) could provide protection against the parental organism. Several different types of vaccines have now been developed. These include: attenuated vaccines rendering targeted microorganisms less toxic and dangerous (e.g., Bacillus Calmette-Guérin and measles), and inactivated vaccines consisting of “killed” microorganisms rendered inoffensive for the host (e.g., *Vibrio cholerae* or *Yersinia pestis*). Further attenuation can come from the use of subunit vaccines which contain only a small fraction of the pathogen. In this case, immunity is based on the antigens requirement for inducing disease or infection (e.g., diphtheria toxin or hepatitis B virus capsular antigen).

Unfortunately, while these approaches have steadily made vaccines safer, killed microorganisms are not always able to trigger efficient immune responses, and toxoid vaccines usually do not trigger long-lasting immune responses. The low cost of attenuated vaccines makes them an attractive vaccination principle; however, such vaccines require the availability of an attenuated strain. Moreover, attenuated vaccines present safety concerns, as they can potentially be harmful for the immune deficient or may revert to wild type (WT) virulence [4].

While the initial vaccine technologies relied on the availability of methods to grow and administer the pathogen in an immunogenic and safe manner, the field of vaccine development has experienced major technological breakthroughs with the advent of recombinant vaccines based on scientific knowledge and molecular biology. A highly versatile new category of vaccines are virus-vectored vaccines, where the knowledge of different organisms allows you to combine the physiology of one organism with the antigen of another. In principle, virus-vectored vaccines could be applied for vaccination against any pathogen. Although acute viral infections tend to provide life-long immunity from a single exposure, a number of limitations have prevented the emergence of vector-based technologies as generalizable and cost-effective vaccination platforms. Different vector types have been explored for vaccination purpose, including influenza virus, lentivirus, adeno-associated virus and yellow fever virus. However, none of these have been found optimal for vaccination use, as they are suboptimal either in stability, production, safety, immunogenicity, or cloning capacity. Finally, adenovirus (Ad) and poxvirus vectors have been widely studied for some of the more challenging antigens (e.g., human immunodeficiency virus (HIV)/malaria).

Both adenoviruses and poxviruses stand out, as they have been used in the most effective vaccine delivery imaginable: replicating vaccinia or adenovirus-vectored vaccines against rabies delivered as bait to wild animals. Adenovirus-based human vaccines have also been developed against adenovirus type 4 and 7, administered as WT virus orally with enteric coating to military recruits to decrease the number of acute respiratory diseases [5]. The military-specific vaccine showed efficiency as well as long-lasting immunity, and was extremely safe with only 2/200,000 developing vaccine-related symptoms [6]. Such a vaccine uses a safely replicating microorganism, delivered in the gut while avoiding the lungs, where it would have caused pathology. Furthermore, it allows in situ dose amplification (lowering production cost), offers easy administration, and leads to the induction of mucosal immunity.

Interestingly, while both replicating poxviruses and adenoviruses have been produced and encompass all the required qualities for an efficient and affordable vaccine, the experience from these vaccines has not been generalized for use against other pathogens.

This review is about where we stand for development of generally applicable tools to design a vaccine offering efficiency, safety, and longevity of protection, using the example of rabies vaccines. We will first discuss the basic properties of vectored vaccines and, in particular, which properties of the rabies vaccine make it so effective. Secondly, we will introduce the case of adenovirus vectors as candidates that can fulfill all requirements for generalized effective vaccination. Finally, we will present some examples where replication-competent adenoviruses have been modified in ways that may render them more alike the rabies virus vaccine gold-standard.

## 2. Vectored Vaccines

### 2.1. Gold Standard, the Example of Rabies Vaccines

As mentioned before, two vectored vaccines against rabies are already in use and encompass all the qualities required for a gold-standard vaccine. The first one is a vaccinia vectored rabies vaccine from the poxvirus family. The construction of the vector was based on the backbone used to eradicate smallpox, inserted with the rabies virus glycoprotein expressed from a poxvirus promoter. The vaccine is delivered orally by placing bait in the wild in order to immunize wildlife animals after ingestion. The vaccinia rabies showed extraordinary results with the targeted species, as well as stability over time and safety for nontargeted species [7]. Moreover, the use of this vaccine led to the elimination of sylvatic rabies from large areas of land in Europe, meaning that vaccination was no longer needed [8].

The company, Artemis Technologies Inc., (Guelph, ON, Canada), has also manufactured an adenovirus 5 for rabies vaccines, designed as a replication-competent human adenovirus type 5 vector with the rabies virus glycoprotein gene inserted in the *E3* region [9]. This new rabies vaccine is stable, effective, and easily administered as bait by aerial distribution [10,11].

The adenovirus rabies vaccine has been tested for a number of wild life species (horses, pigs, sheep, dogs, cats, chickens, meadow voles, foxes, cotton rats, squirrels, rabbits, groundhogs, and cows) and has shown to be perfectly safe for all species and could trigger an antibody response against rabies, in most cases after 28 days [12]. This replication-competent vaccine is effective and safe in animals (also tested for safety in immunocompromised animals) after simple oral administration.

In support of this, several million inoculated bait samples have been distributed in Canada since 2006, and no public safety issue or serious human contact was reported, while local efficiency in eradicating rabies has been described [13].

As the vaccinia vector presents some safety issues due to the incapacity to be sufficiently attenuated for general use in humans, and is relatively poor at inducing T cells, adenoviruses seem to offer some of the best prospects for a generally applicable vaccine platform. Notably, this opinion is supported by the military vaccination campaign, as life-long immunity was induced after single administration (type 4 and 7). The next chapter will be dedicated to a detailed description of adenoviruses and their potential as generally applicable cost-effective vaccine vector candidates.

### 2.2. Lessons Learned from Non-Replicating Adenovirus-Vectored Vaccines

Adenoviruses have been studied as potential vaccine vectors since 1980 [14], and present some of the best results available against cancer, SIV/HIV, herpes virus, papillomavirus, and a number of other diseases [15].

Indeed, adenoviruses have the ability to be propagated to high-titer viral stocks [16], well-established techniques exist for the construction of rAd vectors [17], and they have a well-characterized genome [18,19]. Additionally, adenoviruses have broad cell tropism and can infect a wide spectrum of dividing and nondividing cells with high efficiency of gene transfer [20]. They also induce a potent cellular immune response to encoded antigens, with high numbers of antigens delivered to the lymphoid tissues [21,22]. Moreover, they can encode relatively large DNA inserts compared to many other vector types [23] and WT non-attenuated adenoviral infections are generally without severe clinical symptoms [24].

The infectious cycle of adenoviruses can be divided into two phases. The early phase corresponds to events occurring before initiation of genome amplification: entry of the virus into the host cell and passage of the virus genome to the nucleus. This phase is controlled by the early genes: *E1*, *E2*, *E3*, and *E4*. The *E1* gene is required for inactivating the p53 and retinoblastoma genes and transactivates the other viral promoters in cooperation with *E4*. *E2* is responsible for genome replication, whereas *E3* contains host-specific immune-regulatory genes. The late phase includes assembly of the viral particles in the nucleus and the maturation of infectious virions, and is encoded and controlled by the late genes.

For the simple design of adenovirus-based vaccines, the insertion of foreign genes is based on the deletion of early genes [25], which increases the capacity for inserting foreign antigens and attenuates viral replication.

For safety reasons and convenience, most adenovirus vaccine candidates are *E1* deleted, as *E1* controls the other early genes, thus disabling the expression of early genes. This deletion prevents the virus from spreading in the host and allows selection for recombinant viruses based on replicative capacity in *E1*-expressing cell lines [26]. Δ*E1* adenoviruses have now been used for vaccines in different species such as rodents, nonhuman primates, and humans. Studies have shown that the prototypical replication-deficient adenovirus vector based on the human serotype 5 is well tolerated and strongly immunogenic [27]. Intriguingly, in the case of non-replicative adenovirus vaccines, transgene immunodominance might be facilitated, as the absence of genes controlling replication reduces expression of most adenovirus encoded genes [28].

**Figure 1:**
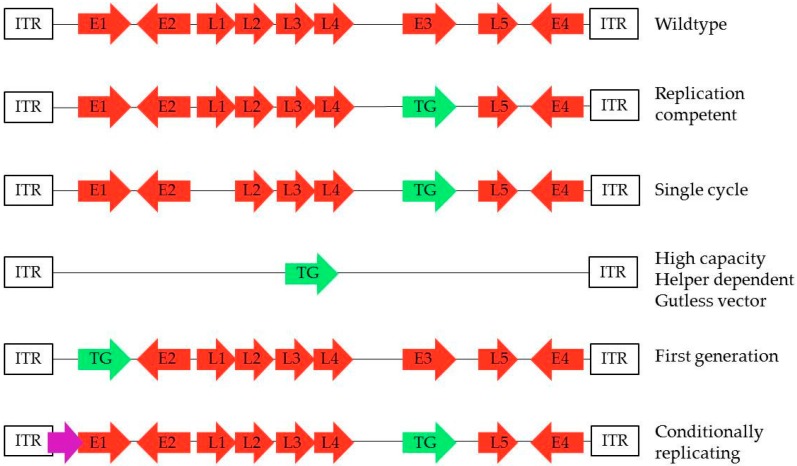
Different possibilities of transgene insertion in the adenoviral genome. The transgene can be inserted in different places in the adenoviral genome, thus offering different characteristics to the adenovirus vector. Transgene can be inserted in place of *E3* gene, leaving *E1* intact together with the capacity of replication, which is the case for most replication-competent (RC), single cycle (SC), and conditionally replicating (CRAd) adenoviruses. The transgene is usually inserted in place of E1, thus preventing replication in the host as seen with first generation (FG). Gutless vectors only contain the ITR domains of the adenovirus and are depleted of all adenoviral genes. These are also called helper-dependent adenoviruses, as they require a helper virus to deliver the capsid genes during production.

However, replication incompetence can only come at the price of lesser immunogenicity, in particular, at low inoculation dosage, as the vaccine self-amplification in vivo is absent. Furthermore, the incapacity of the replication deleted adenovirus to propagate in the host reduces the possibility of reaching the lymphoid organs from mucosal immunization, leading to a requirement for dual route immunization to induce both mucosal and systemic immune responses [29]. Yet, different insertion sites can be considered (see Figure 1), thus offering different properties for the adenovirus vaccine.

As mentioned before, Δ*E1* adenoviruses are inherently suboptimal as vaccine candidates, thus an affordable and cost-effective immunization platform must be based on replication-competent vectors.

## 3. Advantages of Replication-Competent Adenovirus Compared to Other Adenovirus Vectors

Replication competent adenovirus (RCAd) can spread from their inoculation site, efficiently priming responses after injection, and will amplify the originally administered vaccine inoculum. This process can theoretically decrease the cost of the vaccine, as a low injection dose will still allow the adenovirus to self-expand in the host and trigger an immune response towards the in vivo amplified inoculum.

The adenovirus type 5 vector, with the rabies glycoprotein inserted as a replacement of the *E3* gene, is our defined gold-standard, and a lot of different studies have tested the vaccination potential of RCAd against different types of antigens. In this section, we will present some examples showing the capacity of RCAd to induce an efficient immune response against different transgenes.

For vaccination against the HIV envelope, studies in mice revealed that high-capacity adenovirus (HC, also named helper-dependent or gutless vector) [30], devoid of all adenovirus genes, could elicit stronger antibody responses than first generation (FG) vectors [31]. However, in nonhuman primates, where replication of human serotype 5 is possible, replication-competent (RC) vectors were also superior in inducing antibodies and cellular immunity compared to FG vectors [32].

**Figure 2:**
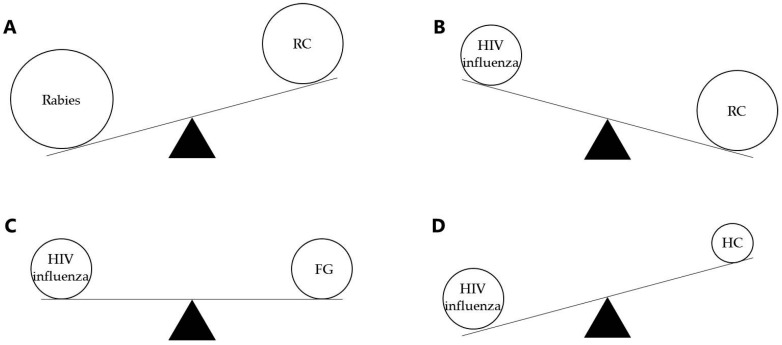
Qualitative comparison of encoded transgene immunodominance as compared to immunodominance induced by the adenoviral vector. Transgenes have different intrinsic immunodominance that competes with different immunodominance of vector antigens in the adenoviral vectors, thus influencing the immune response [37]. As mentioned before, rabies antigens have a really high immunodominance (**A**) as compared to HIV or influenza antigens. The adenoviral vectors also influence the transgene immunodominance offering a strong (**B**), mild (**C**), or low (**D**) competition for the transgene. Thus, antigens like rabies might only require replication-competent adenovirus, but HIV and influenza antigens probably require new adjuvant or other techniques for increased immunodominance, particularly when delivered in replicating vectors.

In both cases, it was or could be speculated that longer presentation of the transgene was the reason for the improved immune responses. In mice, where all compared adenoviruses could not replicate, the removed targets for vector elimination in HC vectors prolonged transgene expression and facilitated the diversity of transgene directed responses [33]. In monkeys, replication competence of the vector results in an extended transgene expression together with an ongoing infectious response [32,34]. The added benefit of robustly stimulating the innate immune system with an RC vector has the disadvantage of the expression of all vectors’ genes and competition with the transgene for induction of immune responses. Since the immunodominance of the vector genes over the transgene is already a problem with FG vaccines [31,33], this problem can only be more pronounced in RC vaccines (Figure 2C).

Despite competition from vector antigens, it appears that the replication competence may still offer a net benefit. Transgene immunogenicity is likely the principal difference between RC rabies vaccine and RC HIV vaccines, as FG rabies vaccine offer potent and long-lasting immune responses after minute doses of adenovirus vaccine [35] (Figure 2A). Influenza-A vaccine, using an envelope target of intermediate immunogenicity, has shown less impressive results when using replication-competent adenoviruses (Figure 2B). For H5N1, RC adenovirus provided a response against H5 in naïve individuals; however, it is expected that a protein boost would be required after priming with RC adenovirus to elicit long-term memory and protection [36].

These different studies have demonstrated greater efficiency and immunogenicity with RC vectors than FG vaccines, by inducing stronger cellular and humoral responses and conferring higher protection, but without approaching the rabies vaccine gold-standard. Whereas the majority of studies are using relatively simple FG or RC adenovirus vector systems, the comparison with complete virus gene-deleted vectors in mice (HC vectors) indicates that vector gene competition is an important parameter [31,33] (Figure 2D). Essentially, the rabies vaccine is, in this review, hypothesized to work by the combination of replication competence and transgene immunodominance. Accordingly, the next two sections will focus on studies which have induced or are anticipated to be capable of increasing transgene immunodominance, either by decreasing vector immunity or by increasing transgene immunogenicity.

## 4. Induction of Transgene Immunodominance by Modulation of the Adenovirus Vector Replication

An alternative to fully replication-competent *E3*-deleted adenoviruses is to selectively modulate the replication capacities of the vector, by genetic mutation or deletion. As we will discuss, modulation of the replication may in principal be used to lower the vector immunodominance as well as offer some level of control over the spread of the adenovirus. Indeed, a major concern about using replication-competent adenovirus is the potential loss of control over replication of the virus, and the resulting potential for severe side-effects in immunocompromised individuals.

### 4.1. Cell- or Tissue-Specific Adenovirus Attenuation

Conditionally replicative adenoviruses (CRAds) have the ability to replicate, but only in a restricted cell type or environment. The use of CRAds could be a crucial step into achieving a cost-effective vaccine standard, as efficiency, safety, and cost-effective qualities potentially can be combined in a single platform.

The classical example of virus attenuation is well established for live attenuated vaccines and consists of temperature attenuation. Development of temperature-sensitive strains seems perfectly doable for adenovirus vectors, but only limited testing for vaccination has been shown [38]. Limitation of virion production in replicative adenovirus is also another way of attenuating the display and accessibility of the adenovirus capsid genes that will no longer be released from lysing cells, while allowing stronger transgene responses from in situ amplified genomes. Single cycle replication-competent adenoviruses (SC), deleted in the penton protein required for virion assembly, have been studied for this purpose. Such viruses were demonstrated to be more immunogenic and allowed a stronger and more persistent transgene expression and specific antibody response than replication-deficient adenoviruses [39]. In the case of influenza, they showed that a 100-fold lower dose of a single cycle replication-competent adenovirus was able to induce higher level of antibodies, compared to replication deficient adenovirus [40].

To ensure a cell-specific replication profile, the *E1* genes, responsible for expression of the early genes, can be placed under the control of a cell-specific promoter, thus restricting the replication only in a selected cell type. This method has been tested using a large range of different promoters, as the promoter has to be chosen considering the vaccination route and the disease to target. As an example, this method could in principle be applied to limit replication to specific cells such as epithelial cells (e.g., allowing oral administration) or antigen-presenting cells.

CRAds have mostly been studied for cancer gene therapy, where the *E1* gene is placed under the control of a tumor-specific cell promoter (reviewed by Curiel in [41]), and have shown high specificity in replication, but limited anticancer efficacy. Such viruses have been used to target and limit replication to cancer cells and used the capacity of the virus to lyse and kill infected cells. Cell-specific inhibition of replication or expression of virus genes have also been studied to a lesser extent, using cell-specific miRNA, in the case of influenza A virus [42], hepatitis C virus [43], and also adenovirus [44].

**Figure 3:**
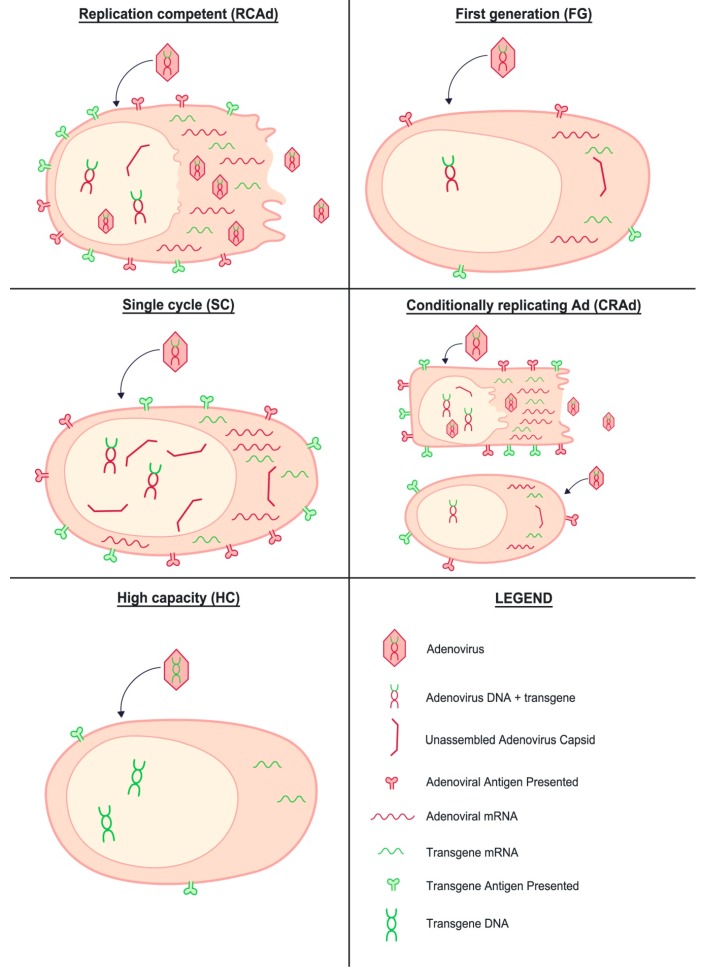
Overview of the quantitative vector and transgene expression using different adenovirus platforms for vaccination.

Although, in the case of CRAds, the use of cell-specific replication can be used to lower the vector immunogenicity and improve the safety of the vaccine, it is equally important to increase the expression of the transgene (see Figure 3, which qualitatively depicts the differential expression of transgene and vector for the different adenovirus candidates discussed in the review). It is noteworthy that in the majority of RC adenovirus vaccines, the transgene expression is coupled to the virus replication and occurs from the *E3* reading frame. As the *E3* gene is turned on later than the *E1* and *E4* reading frames, expression of the transgene through the *E3* promoter [45] does not offer the most optimal design for inducing transgene-specific immune responses. It is a testament to this consideration that the most effective improvements of adenovirus immunogenicity have been the SC-adenovirus vaccines, where a heterologous promoter was used to direct transgene expression. For fully RC vaccines, it is of crucial importance to realize that the principle of (cell-specific) virus attenuation is to limit vector replication, but it would be detrimental to simultaneously limit transgene expression. As an example, temperature-sensitive adenovirus could replicate in the upper airways and reach dendritic cells, but then suffer undesirable attenuation of transgene expression in antigen-presenting cells in draining lymph nodes. The use of two different promoters to direct transgene expression and replication as performed to track oncolytic viruses [46] or an exogenous promoter for antigen in temperature-sensitive viruses would eliminate this problem. In this case, while replication of the adenovirus would be limited to specific cells, the expression of the transgene will be extended to all cells, ensuring a long-lasting presentation of the transgene to the immune system. This strategy could promote a stronger immunodominance of the transgene over the vector, while keeping some of the advantages of using replication-competent adenovirus, as described above.

### 4.2. Increase of Transgene Immunodominance

Immunodominance of the transgene can in general be improved by using stronger promoters [47,48,49] or by using specific adjuvants genetically linked to the transgene. As an example, the invariant chain (II) of the MHCII was shown to be a surprisingly effective T cell inducer when used as an adjuvant in the case of adenoviruses. Indeed, when the transgene was genetically coupled to the invariant chain, it showed a consistent enhancement in CD8+ and CD4+ T cell responses as well as longer-lasting immune responses [50,51,52]. Using the same strategy, the oligomerization domain of C4b was also capable of increasing the T cell response against the transgene [53], as well as the antibody response against malaria when used as an adjuvant in adenovirus vaccines [54].

T cell responses are usually quite high after adenovirus vaccination, though B cell responses targeting the transgene might need to be improved after single adenoviral immunization. Indeed, when the transgene is encoded in the adenoviral genome, it is mostly presented to the immune system through MHCI; however, B cells are mostly activated after presenting the antigen to CD4+ T cells via the MHCII. Different methods have increased the humoral response after incorporation of transgene in to the different capsid proteins of the adenovirus compared to encoded antigen [55]. As hexon is the most abundant capsid protein, it is expected that incorporation of antigens in the hexon protein would yield the most antigen-specific immune responses [56]. Unfortunately, fiber and hexon only allow incorporation of small peptides. However, pIX tolerates incorporation of large proteins, allowing presentation of conformational antigens to the immune system, leading to a broader immune responses as well as activation of conformational epitope for B cells’ activation [57]. The pIX immunogenicity can further be improved by combination of display and transgene expression of antigen. This technique has been taken quite far in the example of adenovirus that encoded and displayed antigen on the surface of a replicating viral vaccine [58].

Increase of transgene-specific B cell responses can also be achieved by encoding virus-like particles (VLPs) within the adenovirus genome and having these particles display the antigen. In this case, the infected cell will produce VLPs that will present the transgene on their outer membrane. Adenovirus-encoded VLPs—using SIV gag and a recombinant plasmodial antigen fused to a viral envelope transmembrane tail—elicited an increased level of antibody responses and, most importantly, showed better functionality of the induced antibodies [59]. This could potentially be explained by the fact that conformational antigens are presented directly to B cells from secreted VLPs, thus resembling a normal infection, and inducing conformational antibodies required for effective neutralization. 

## 5. Conclusions

Knowledge about vaccine strategies has increased over the years and access to vaccination has been enhanced around the world; this has permitted the extinction of some diseases. However, the requirements for even better vaccines to fight more complex diseases (HIV, malaria, cancer) but also for more accessible vaccines (low cost and easy administration) have not been met. Such vaccines exist in particular cases (e.g., Ad4 and 7 in military recruits, rabies in wildlife), but tools for their generally applicable development and use have not been established yet. In order to produce very affordable, yet effective, vaccines, a requirement would be a cell-specific replicating vaccine which could increase the recombinant transgene-directed immune response, as suggested to be possible by some studies. Most importantly, with replicating vaccines, a low inoculation dose would confer sufficient immune response due to amplification at the immunization site, while restricted replication only in targeted cells or tissues would increase safety and potentially immune focus on the transgene. Conditionally replicating adenoviruses also permit mucosal administration, offering mucosal immune responses (required against most infectious diseases), as well as allowing easy administration through aerosol sprays or tablets. Technologies may already exist to enable such improvements, but they have yet to be combined and tested for vaccination approaches.

## Abbreviations

Ad:	Adenovirus
CRAd:	Conditionally replicating adenovirus
DNA:	Deoxyribonucleic-acid
FG:	First generation
HC:	High capacity
HIV:	Human immunodeficiency virus
MHC:	Major histocompatibility complex
RC:	Replication competent
RCAd:	Replication competent adenovirus
RNA:	Ribonucleic acid
VLP:	Virus like particle
WT:	Wild-type

## References

[1] Andre F.E., Booy R., Bock H.L., Clemens J., Datta S.K., John T.J., Lee B.W., Lolekha S., Peltola H., Ruff T.A. (2008). Vaccination greatly reduces disease, disability, death and inequity worldwide. Bull. World Health Organ..

[2] Folb P.I., Bernatowska E., Chen R., Clemens J., Dodoo A.N., Ellenberg S.S., Farrington C.P., John T.J., Lambert P.H., Macdonald N.E. (2004). A global perspective on vaccine safety and public health: The Global Advisory Committee on Vaccine Safety. Am. J. Public Health.

[3] Zhou W., Pool V., Iskander J.K., English-Bullard R., Ball R., Wise R.P., Haber P., Pless R.P., Mootrey G., Ellenberg S.S. (2003). Surveillance for safety after immunization: Vaccine Adverse Event Reporting System (VAERS)—United States, 1991–2001. MMWR Surveill. Summ..

[4] Shimizu H., Thorley B., Paladin F.J., Brussen K.A., Stambos V., Yuen L., Utama A., Tano Y., Arita M., Yoshida H. (2004). Circulation of type 1 vaccine-derived poliovirus in the Philippines in 2001. J. Virol..

[5] Artenstein A.W., Opal J.M., Opal S.M., Tramont E.C., Peter G., Russell P.K. (2005). History of U.S. military contributions to the study of vaccines against infectious diseases. Mil. Med..

[6] Choudhry A., Mathena J., Albano J.D., Yacovone M., Collins L. (2016). Safety evaluation of adenovirus type 4 and type 7 vaccine live, oral in military recruits. Vaccine.

[7] Pastoret P.P., Brochier B. (1996). The development and use of a vaccinia-rabies recombinant oral vaccine for the control of wildlife rabies; a link between Jenner and Pasteur. Epidemiol. Infect..

[8] Brochier B., Aubert M.F., Pastoret P.P., Masson E., Schon J., Lombard M., Chappuis G., Languet B., Desmettre P. (1996). Field use of a vaccinia-rabies recombinant vaccine for the control of sylvatic rabies in Europe and North America. Rev. Sci. Tech..

[9] Yarosh O.K., Wandeler A.I., Graham F.L., Campbell J.B., Prevec L. (1996). Human adenovirus type 5 vectors expressing rabies glycoprotein. Vaccine.

[10] Rosatte R.C., Donovan D., Davies J.C., Allan M., Bachmann P., Stevenson B., Sobey K., Brown L., Silver A., Bennett K. (2009). Aerial distribution of ONRAB baits as a tactic to control rabies in raccoons and striped skunks in Ontario, Canada. J. Wildl. Dis..

[11] Ontario Resuming Rabies Vaccination Bait Drops. https://news.ontario.ca/mnr/en/2016/03/ontario-resuming-rabies-vaccination-bait-drops.html.

[12] Knowles M.K., Nadin-Davis S.A., Sheen M., Rosatte R., Mueller R., Beresford A. (2009). Safety studies on an adenovirus recombinant vaccine for rabies (AdRG1.3-ONRAB) in target and non-target species. Vaccine.

[13] Rosatte R.C., Donovan D., Davies J.C., Brown L., Allan M., von Zuben V., Bachmann P., Sobey K., Silver A., Bennett K. (2011). High-density baiting with ONRAB(R) rabies vaccine baits to control Arctic-variant rabies in striped skunks in Ontario, Canada. J. Wildl. Dis..

[14] Alkhatib G., Briedis D.J. (1988). High-level eucaryotic in vivo expression of biologically active measles virus hemagglutinin by using an adenovirus type 5 helper-free vector system. J. Virol..

[15] Lasaro M.O., Ertl H.C. (2009). New insights on adenovirus as vaccine vectors. Mol. Ther..

[16] Kamen A., Henry O. (2004). Development and optimization of an adenovirus production process. J. Gene Med..

[17] Danthinne X., Imperiale M.J. (2000). Production of first generation adenovirus vectors: A review. Gene Ther..

[18] Randrianarison-Jewtoukoff V., Perricaudet M. (1995). Recombinant adenoviruses as vaccines. Biologicals.

[19] Imler J.L. (1995). Adenovirus vectors as recombinant viral vaccines. Vaccine.

[20] Vorburger S.A., Hunt K.K. (2002). Adenoviral gene therapy. Oncologist.

[21] Yang T.C., Dayball K., Wan Y.H., Bramson J. (2003). Detailed analysis of the CD8+ T-cell response following adenovirus vaccination. J. Virol..

[22] Vanniasinkam T., Ertl H.C. (2005). Adenoviral gene delivery for HIV-1 vaccination. Curr. Gene Ther..

[23] Bett A.J., Prevec L., Graham F.L. (1993). Packaging capacity and stability of human adenovirus type 5 vectors. J. Virol..

[24] Ferreira T.B., Alves P.M., Aunins J.G., Carrondo M.J. (2005). Use of adenoviral vectors as veterinary vaccines. Gene Ther..

[25] Haj-Ahmad Y., Graham F.L. (1986). Development of a helper-independent human adenovirus vector and its use in the transfer of the herpes simplex virus thymidine kinase gene. J. Virol..

[26] Russell W.C. (2000). Update on adenovirus and its vectors. J. Gen. Virol..

[27] Liniger M., Zuniga A., Naim H.Y. (2007). Use of viral vectors for the development of vaccines. Expert Rev. Vaccines.

[28] Schagen F.H., Ossevoort M., Toes R.E., Hoeben R.C. (2004). Immune responses against adenoviral vectors and their transgene products: A review of strategies for evasion. Crit. Rev. Oncol. Hematol..

[29] Nazerai L., Bassi M.R., Uddback I.E., Holst P.J., Christensen J.P., Thomsen A.R. (2016). Early life vaccination: Generation of adult-quality memory CD8+ T cells in infant mice using non-replicating adenoviral vectors. Sci. Rep..

[30] Kochanek S. (1999). High-capacity adenoviral vectors for gene transfer and somatic gene therapy. Hum. Gene Ther..

[31] Weaver E.A., Nehete P.N., Buchl S.S., Senac J.S., Palmer D., Ng P., Sastry K.J., Barry M.A. (2009). Comparison of replication-competent, first generation, and helper-dependent adenoviral vaccines. PLoS ONE.

[32] Peng B., Wang L.R., Gomez-Roman V.R., Davis-Warren A., Montefiori D.C., Kalyanaraman V.S., Venzon D., Zhao J., Kan E., Rowell T.J. (2005). Replicating rather than nonreplicating adenovirus-human immunodeficiency virus recombinant vaccines are better at eliciting potent cellular immunity and priming high-titer antibodies. J. Virol..

[33] Kron M.W., Engler T., Schmidt E., Schirmbeck R., Kochanek S., Kreppel F. (2011). High-capacity adenoviral vectors circumvent the limitations of ΔE1 and ΔE1/ΔE3 adenovirus vectors to induce multispecific transgene product-directed CD8 T-cell responses. J. Gene Med..

[34] Demberg T., Florese R.H., Heath M.J., Larsen K., Kalisz I., Kalyanaraman V.S., Lee E.M., Pal R., Venzon D., Grant R. (2007). A replication-competent adenovirus-human immunodeficiency virus (Ad-HIV) tat and Ad-HIV env priming/Tat and envelope protein boosting regimen elicits enhanced protective efficacy against simian/human immunodeficiency virus SHIV89.6P challenge in rhesus macaques. J. Virol..

[35] Wang Y., Xiang Z., Pasquini S., Ertl H.C. (1997). The use of an E1-deleted, replication-defective adenovirus recombinant expressing the rabies virus glycoprotein for early vaccination of mice against rabies virus. J. Virol..

[36] Khurana S., Coyle E.M., Manischewitz J., King L.R., Ishioka G., Alexander J., Smith J., Gurwith M., Golding H. (2015). Oral priming with replicating adenovirus serotype 4 followed by subunit H5N1 vaccine boost promotes antibody affinity maturation and expands H5N1 cross-clade neutralization. PLoS ONE.

[37] Pinschewer D.D., Perez M., Jeetendra E., Bachi T., Horvath E., Hengartner H., Whitt M.A., de la Torre J.C., Zinkernagel R.M. (2004). Kinetics of protective antibodies are determined by the viral surface antigen. J. Clin. Invest..

[38] Zygraich N., Lobmann M., Peetermans J., Vascoboinic E., Huygelen C. (1975). Local and systemic response after simultaneous intranasal inoculation of temperature-sensitive mutants of parainfluenza 3, IBR and bovine adenovirus 3. Dev. Biol. Stand..

[39] Crosby C.M., Nehete P., Sastry K.J., Barry M.A. (2015). Amplified and persistent immune responses generated by single-cycle replicating adenovirus vaccines. J. Virol..

[40] Crosby C.M., Matchett W.E., Anguiano-Zarate S.S., Parks C.A., Weaver E.A., Pease L.R., Webby R.J., Barry M.A. (2017). Replicating Single-Cycle Adenovirus Vectors Generate Amplified Influenza Vaccine Responses. J. Virol..

[41] Curiel D.T. (2000). The development of conditionally replicative adenoviruses for cancer therapy. Clin. Cancer Res..

[42] Langlois R.A., Albrecht R.A., Kimble B., Sutton T., Shapiro J.S., Finch C., Angel M., Chua M.A., Gonzalez-Reiche A.S., Xu K. (2013). MicroRNA-based strategy to mitigate the risk of gain-of-function influenza studies. Nat. Biotechnol..

[43] Mizuguchi Y., Takizawa T., Uchida E. (2015). Host cellular microRNA involvement in the control of hepatitis B virus gene expression and replication. World J. Hepatol..

[44] Ylosmaki E., Hakkarainen T., Hemminki A., Visakorpi T., Andino R., Saksela K. (2008). Generation of a conditionally replicating adenovirus based on targeted destruction of E1A mRNA by a cell type-specific MicroRNA. J. Virol..

[45] Mei Y.F., Wu H., Hultenby K., Silver J. (2016). Complete replication-competent adenovirus 11p vectors with E1 or E3 insertions show improved heat stability. Virology.

[46] Davis J.J., Wang L., Dong F., Zhang L., Guo W., Teraishi F., Xu K., Ji L., Fang B. (2006). Oncolysis and suppression of tumor growth by a GFP-expressing oncolytic adenovirus controlled by an hTERT and CMV hybrid promoter. Cancer Gene Ther..

[47] Richardson J.S., Yao M.K., Tran K.N., Croyle M.A., Strong J.E., Feldmann H., Kobinger G.P. (2009). Enhanced protection against Ebola virus mediated by an improved adenovirus-based vaccine. PLoS ONE.

[48] Sridhar S., Reyes-Sandoval A., Draper S.J., Moore A.C., Gilbert S.C., Gao G.P., Wilson J.M., Hill A.V. (2008). Single-dose protection against Plasmodium berghei by a simian adenovirus vector using a human cytomegalovirus promoter containing intron A. J. Virol..

[49] Alharbi N.K., Spencer A.J., Salman A.M., Tully C.M., Chinnakannan S.K., Lambe T., Yamaguchi Y., Morris S.J., Orubu T., Draper S.J. (2016). Enhancing cellular immunogenicity of MVA-vectored vaccines by utilizing the F11L endogenous promoter. Vaccine.

[50] Spencer A.J., Cottingham M.G., Jenks J.A., Longley R.J., Capone S., Colloca S., Folgori A., Cortese R., Nicosia A., Bregu M. (2014). Enhanced vaccine-induced CD8+ T cell responses to malaria antigen ME-TRAP by fusion to MHC class ii invariant chain. PLoS ONE.

[51] Holst P.J., Sorensen M.R., Mandrup Jensen C.M., Orskov C., Thomsen A.R., Christensen J.P. (2008). MHC class II-associated invariant chain linkage of antigen dramatically improves cell-mediated immunity induced by adenovirus vaccines. J. Immunol..

[52] Ragonnaud E., Andersson A.M., Pedersen A.E., Laursen H., Holst P.J. (2016). An adenoviral cancer vaccine co-encoding a tumor associated antigen together with secreted 4-1BBL leads to delayed tumor progression. Vaccine.

[53] Forbes E.K., de Cassan S.C., Llewellyn D., Biswas S., Goodman A.L., Cottingham M.G., Long C.A., Pleass R.J., Hill A.V., Hill F. (2012). T cell responses induced by adenoviral vectored vaccines can be adjuvanted by fusion of antigen to the oligomerization domain of C4b-binding protein. PLoS ONE.

[54] Li Y., Leneghan D.B., Miura K., Nikolaeva D., Brian I.J., Dicks M.D., Fyfe A.J., Zakutansky S.E., de Cassan S., Long C.A. (2016). Enhancing immunogenicity and transmission-blocking activity of malaria vaccines by fusing Pfs25 to IMX313 multimerization technology. Sci. Rep..

[55] Matthews Q.L. (2011). Capsid-incorporation of antigens into adenovirus capsid proteins for a vaccine approach. Mol. Pharm..

[56] Krause A., Joh J.H., Hackett N.R., Roelvink P.W., Bruder J.T., Wickham T.J., Kovesdi I., Crystal R.G., Worgall S. (2006). Epitopes expressed in different adenovirus capsid proteins induce different levels of epitope-specific immunity. J. Virol..

[57] Meulenbroek R.A., Sargent K.L., Lunde J., Jasmin B.J., Parks R.J. (2004). Use of adenovirus protein IX (pIX) to display large polypeptides on the virion—Generation of fluorescent virus through the incorporation of pIX-GFP. Mol. Ther..

[58] Karen K.A., Deal C., Adams R.J., Nielsen C., Ward C., Espinosa D.A., Xie J., Zavala F., Ketner G. (2015). A replicating adenovirus capsid display recombinant elicits antibodies against Plasmodium falciparum sporozoites in Aotus nancymaae monkeys. Infect. Immun..

[59] Andersson A.C., Resende M., Salanti A., Nielsen M.A., Holst P.J. (2017). Novel adenovirus encoded virus-like particles displaying the placental malaria associated VAR2CSA antigen. Vaccine.

